# Gas-Chromatography Mass-Spectrometry (GC-MS) Based Metabolite Profiling Reveals Mannitol as a Major Storage Carbohydrate in the Coccolithophorid Alga *Emiliania huxleyi.*

**DOI:** 10.3390/metabo3010168

**Published:** 2013-03-11

**Authors:** Toshihiro Obata, Steffi Schoenefeld, Ina Krahnert, Susan Bergmann, André Scheffel, Alisdair R. Fernie

**Affiliations:** Max-Planck-Institut für Molekulare Pflanzenphysiologie, Potsdam-Golm 14476, Germany; E-Mails: schoenefeld@mpimp-golm.mpg.de (S.S.); krahnert@mpimp-golm.mpg.de (I.K.); bergmann@mpimp-golm.mpg.de (S.B.); scheffel@mpimp-golm.mpg.de (A.S.); fernie@mpimp-golm.mpg.de (A.R.F.)

**Keywords:** coccolithophorid, *Emiliania huxleyi*, metabolite profiling, GC-MS, primary carbon metabolism, mannitol, isotope labelling

## Abstract

Algae are divergent organisms having a wide variety of evolutional histories. Although most of them share photosynthetic activity, their pathways of primary carbon metabolism are rather diverse among species. Here we developed a method for gas chromatography-mass spectroscopy (GC-MS) based metabolite profiling for the coccolithophorid alga *Emiliania huxleyi*, which is one of the most abundant microalgae in the ocean, in order to gain an overview of the pathway of primary metabolism within this alga. Following method optimization, twenty-six metabolites could be detected by this method. Whilst most proteogenic amino acids were detected, no peaks corresponding to malate and fumarate were found. The metabolite profile of *E*. *huxleyi* was, however, characterized by a prominent accumulation of mannitol reaching in excess of 14 nmol 10^6^ cells^−1^. Similarly, the accumulation of the ^13^C label during short term H^13^CO_3_^−^ feeding revealed a massive redistribution of label into mannitol as well as rapid but saturating label accumulation into glucose and several amino acids including aspartate, glycine and serine. These results provide support to previous work suggesting that this species adopts C_3_ photosynthesis and that mannitol functions as a carbon store in *E*. *huxleyi*.

## 1. Introduction

Algae are divergent organisms derived from multiple endosymbiosis events. Eukaryotic photoautotrophs emerged by primary endosymbiosis during which a eukaryotic cell acquired cyanobacterium as a plastid. Secondary endosymbiosis whereby a non-photosynthetic eukaryote acquired a plastid by engulfing a photosynthetic eukaryote gave rise to diverse algal lineages [1]. In each endosymbiotic event, the genes from the plastid and the host are sorted out to establish a distinctive gene and metabolic networks. In addition there is accumulating evidence of horizontal gene transfer among marine organisms especially from bacteria to algae [2,3] by means of which algae could involve new enzymes into their metabolic networks. Such an evolutional background renders algae as "melting pots" of genes and provides them with an unprecedented opportunity to evolve both novel genetic and metabolite networks [4]. This opportunity is reflected in the diversity of central carbon metabolisms among algal species. One such example is that alga possesses diverse storage polysaccharide including α- (starch) and β-glucans (laminarin, chrysolaminarin and paramylon), which has been used as diagnostic trait for taxonomic analysis [5]. β-glucans are present in highly abundant algae such as coccolithophores and diatoms rendering it vital in terms of carbon and energy fluxes in marine habitats [6]. However, metabolic pathways related to β-glucans remain unclear in contrast to the biosynthetic pathways of starch, which have been well characterized in plants and green algae. Greater understanding of the metabolic pathways and their constituent enzymes will likely not only be of fundamental importance to understand their diverse physiology but will also likely facilitate metabolic engineering or direct industrial processing of these species.

Gas chromatography-mass spectrometry (GC-MS) based metabolite profiling is a useful tool to gain an overview of central carbon metabolism since it facilitates the identification and robust quantification of up to 100 metabolites in simple plant extracts. These metabolites include sugars, sugar alcohols, amino acids, organic acids and polyamines, resulting in fairly comprehensive coverage of the central pathways of primary metabolism [7]. Nevertheless, the use of this technique in algal studies is still relatively rare [4]. This is at least partly due to the complexity of establishing robust sampling and extraction procedures for these species since each algal species has different mechanical and chemical stabilities and as such requires a specialized extraction procedure. For example three species of cyanobacteria required different length of sonication and incubation for complete extraction of metabolites [8]. Here we describe the establishment of the method for GC-MS analysis of the coccolithophorid alga, *Emiliania huxleyi*. Coccolithophorids are unicellular algae of the division haptophyta characterized by bearing an extracellular coat of calcareous plates, coccoliths. They are considered as main producers of biogenic calcium carbonate in the oceans and a significant source of dimethylsulfoniopropionate, which is a precursor of the climatically important gas dimethylsulfide [9]. *E . huxleyi* is the dominant and most widely-spread coccolithophore in contemporary oceans and capable of forming vast blooms [10,11]. The metabolic network underlying the successful physiological adaptation to a wide range of environmental conditions, however, remains mostly elusive. This alga most likely possesses a distinctive metabolic network since some reports have already suggested the presence of unusual metabolic pathways in it. For example, a series of studies indicate that *E*. *huxleyi* possesses unique carbon assimilation mechanisms involving β-carboxylation by both pyruvate carboxylase and phospho*enol*pyruvate carboxylase in different organelles [12,13]. It has also been suggested that *E*. *huxleyi* operates distinctive metabolic pathways in order to achieve efficient use of the essential micronutrient selenium [14,15,16]. GC-MS based metabolite profiling would be a useful tool to uncover the primary metabolic network in this alga by combining the results with genomic sequence information available [17]. In the current study we established a procedure for sample preparation for GC-MS analyses and applied ^13^C-label redistribution techniques in order to gain an overview of the CO_2_ fixation pathway in *E*. *huxleyi*. The results suggested an important role of mannitol as storage carbohydrate, and hence a distinctive CO_2_ fixation pathway in this alga is proposed.

## 2. Results and Discussion

### 2.1. Optimization of Sample Preparation Protocol for E. huxleyi

Here we aimed to establish sampling and extraction protocols for GC-MS analysis to explore relatively abundant metabolites in a wide range of growth conditions. To ensure a rapid sampling from the culture with various cell densities, it is necessary to choose an appropriate sampling method. Centrifugation and filtration are the first choices for collecting the cells of unicellular algae. Direct quenching in cold organic solvent is also applicable for GC-MS analysis. Centrifugation and direct quenching are suitable to collect algal cells from up to several milliliters of culture solution. However, we could not collect enough biomass from the small volume of *E*. *huxleyi* culture even in the late logarithmic growth phase due to the relatively low cell density compared to organisms such as *Chlamydomonas reinhardtii* and cyanobacteria. By vacuum filtration the cells in 10 ml of culture could readily be collected within 10 s. Therefore, we chose filtration to keep open the possibility of applying the method to the samples with low cellular density, for instance those from the early stages of batch cultures. Another reason to choose filtration was that some *E*. *huxleyi* cells stuck to the sides of the tubes following centrifugation which could be anticipated as a source of experimental error. Thus filtration should be the choice for metabolite profiling not only for *E*. *huxleyi* but also for many other algae, which do not grow to a high cell density.

Secondly, we optimized the extraction procedure. We adopted a well-established protocol for extracting metabolites from cyanobacteria on a filter described in [8]. To test the effect of incubation time on the efficiency of metabolite extraction from *E*. *huxleyi*, the cells were incubated in 90% methanol at 4 °C for 0, 30 and 60 min after vortex, and chlorophyll *a* (Chl*a*) in the supernatant was determined as an index of extracted metabolites (Supplementary Figure 1a). The extracted Chl*a* level was unaltered following 30 min of incubation, but slightly decreased following 60 min of incubation even when samples were processed under dim light. This suggests that a long incubation can increase the risk of metabolite degradation. The effect of sonication was also tested. Cells were sonicated three times for 1 min in ice-cold water after vortex. The Chl*a* extraction was examined after vortex, sonication and following 60 min incubation. In this experiment no change was observed in Chl*a* amount after sonication and incubation (Supplementary Figure 1b). Following both experiments, cell debris was collected and extracted in 90% methanol again after washing with the same solution. No Chl*a* was detected in these extracts indicating that all Chl*a* was extracted during the experiments. These studies indicate that the addition of 90% methanol and vortex are sufficient to extract small polar metabolites for GC-MS analysis.

**Figure 1:**
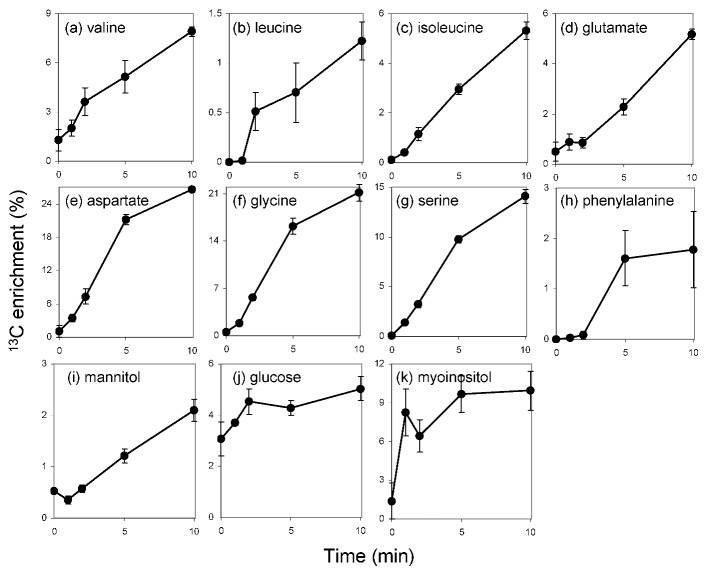
Time course of ^13^C-isotope enrichment in the metabolites during 10 min of H^13^CO_3_^−^ feeding. Note that the enrichment values are different in each metabolite. Values are means of percentages of ^13^C-labeled mass fragments ± standard error of means from four biological replicates.

In a next step, the approximate amount of cells in order to maximize the number of detected metabolites was determined. We used the cell suspension in the late logarithmic growth phase in which the cell density was 2.32 × 10^6^ cells ml^−1^. This cell density had the following properties, optical density at 750 nm (OD_750_) of 0.26, cell volume of 0.94 µL cells mL^−1^ and 1.38 µg Chl*a* mL^−1^. We collected 0.5, 1.0, 2.5, 5.0 and 10.0 ml of the culture including 1.16, 2.32, 5.80, 11.6 and 23.8 × 10^6^ cells, respectively. Samples were analyzed by GC-MS and the number of detected metabolites was determined. A metabolite was counted as detected when the corresponding peak could be identified and the level of the metabolite was positive after background subtraction in at least three of four replicates. The linear relationship between the cell number and the level of metabolite was also considered to distinguish if it was a genuine metabolite or a contaminant. The number of detected metabolites was increased in relation to cellular amount up to 11.6 × 10^6^ cells and then saturated. In the sample with 23.8 × 10^6^ cells, two metabolites were overloaded (Table 1, Supplementary Table 1). This suggests that the most suitable amount of cells for the metabolite profiling of *E*. *huxleyi* is around 1.0 × 10^7^, which corresponds to 1.25 OD_750_ ml, 5.0 µL of cell volume and 6.75 µg of Chl*a*. Further increase of cell amount may help to detect low abundant metabolites such as minor amino acids, organic acids and sugars. However we settled on this amount since it allows quantitative analysis of the most abundant metabolites, and some metabolites started to saturate with higher amount of cells (Supplementary Table 1, Table 1). Additionally higher collection volume of culture solution impairs rapid sampling, which is favorable for the analysis of unstable metabolites.

**Table 1:** Metabolites identified in *E*. *huxleyi* cell extract by gas chromatography-mass spectroscopy (GC-MS) analysis. Cell number represents the smallest amount of the cells needed to detect the metabolites. 1, 2, 5, 10 and 20 correspond to 1.16, 2.32, 5.80, 11.6 and 23.2 × 10^6^ cells, respectively. Metabolites with asterisk were overloaded in the samples of 23.2 × 10^6^ cells.

Metabolite	Cell number
Amino acids	
Isoleucine	2
Valine	2
Leucine	5
Threonine	5
Serine	5
Alanine	10
Aspartate	10
Glutamate	10
Glycine	10
Lysine	10
*O*-acetylserine	10
Phenylalanine	10
Organic acids	
Dehydroascorbate	1
Citrate	5
Glycerate	5
Threonate	5
Fatty acids	
Dodecanoate*	2
Decanoate	5
Sugars and sugar alcohols	
Mannitol*	1
Glucose	2
myo-inositol	2
Ribose	2
Sucrose	2
Maltotriose	5
Fructose	10
Tocopherol	
*α*-tocopherol	10

We also performed recombination experiments to examine the robustness of the method. Extracts of *E*. *huxleyi* and Arabidopsis were subjected to GC-MS investigations, both in isolation and then as a stoichiometric mixture. This serves two purposes. Firstly it validates that the peak identification for *E*. *huxleyi* samples is the same as that for Arabidopsis leaves. This experiment is of particular importance given that different extract compositions can cause so-called “matrix effects” that can result in shifts in relative elution times [18]. Whilst these rarely cause problems for peak annotation in the case of GC-MS, the possibility cannot simply be ignored. Most of the metabolites were detected in the mixed samples except for two very minor metabolites in *E*. *huxleyi*, citrate and *O*-acetylserine. This suggests that matrix effects did not cause problems with peak annotation in *E*. *huxleyi*. Using the same experimental results, we evaluated whether the relative values determined in the simple algal extracts could be quantifiably retrieved in the mixed extract (Table 2). Eighteen of twenty-four metabolites that we evaluated in this manner were recovered at between 70% and 130% of the level at which they were added. The quantitative results on other metabolites should be considered with special care especially when they are compared with other organisms.

**Table 2:** Quantitative recovery of *E*. *huxleyi* metabolites following recombination with Arabidopsis leaf extract. Values are presented as the means ± standard error of means from four technical replicates. Citrate and *O*-acetylserine could not be determined in this measurement due to very low content.

Metabolite	Recovery in %
Alanine	87.79 ± 1.55
Aspartate	34.11 ± 1.27
Decanoate	149.30 ± 4.71
Dehydroascorbate	86.38 ± 1.91
Dodecanoate	121.54 ± 11.23
Fructose	82.47 ± 24.30
Glucose	166.95 ± 14.08
Glutamate	53.93 ± 3.49
Glycerate	94.042 ± 3.86
Glycine	91.64 ± 2.84
Isoleucine	94.63 ± 2.93
Leucine	89.90 ± 1.94
Lysine	73.82 ± 3.48
Maltotriose	65.45 ± 0.93
Mannitol	102.87 ± 3.97
Myoinositol	88.40 ± 2.60
Phenylalanine	71.02 ± 2.53
Ribose	129.13 ± 5.13
Serine	64.95 ± 2.19
Sucrose	106.86 ± 4.52
Threonate	88.34 ± 4.52
Threonine	81.14 ± 2.17
Valine	97.26 ± 1.98
alpha-tocopherol	71.97 ± 3.40

### 2.2. Metabolite Profiling Revealed Metabolic Features of E. huxleyi

Using the optimized protocol, the metabolite profile of *E*. *huxleyi* cells in late logarithmic growth phase was analyzed. Twenty-six annotated metabolites are listed in Table 1. They include major amino acids, some organic acids, sugars, polyols, fatty acids and a tocopherol. The result highlights some interesting metabolic features of *E*. *huxleyi*.

#### 2.2.1. High Mannitol Content and Faint Amounts of Fructose and Sucrose

Mannitol was the most prominent peak of the chromatogram, which was detected from 1 × 10^6^ cells and was overloaded in the sample of 20 × 10^6^ cells (Table 1). It was much more prominent than the peaks of sugars such as fructose, glucose and sucrose which are abundant in most green plants. The absolute amount of mannitol reached to 14.1 nmol 10^6^ cells^−1^. Even when the equal distribution in the cell was assumed, the mannitol concentration was calculated as 34.8 mM. The actual concentration should be higher given that cytosolic localization of mannitol has been reported in some organisms [19,20,21]. Mannitol is a polyol most widely spread in photosynthetic organisms [19]. Whilst mannitol is hardly detected in many green plants including Arabidopsis, it composes a significant portion of soluble carbohydrate in the species of Oleaceae (olive, privet), Apiaceae (celery, carrot, parsley) and Rubiaceae (coffee) [21]. In many photosynthetic organisms including brown algae, mannitol is synthesized as a major primary photosynthetic product [22] and is used as an important translocatory [23] and storage compound [24]. Mannitol also serves as important osmolyte and compatible solute, providing tolerance to salinity in higher plants [21]. Such high accumulation of mannitol suggests the role of mannitol as storage compound in *E*. *huxleyi*.

#### 2.2.2. Detection of Antioxidants

Certain peaks of dehydroascorbate were detected even from the samples of smallest amount of cells (Table 1). Since ascorbate should be oxidized during the metabolite extraction, accumulation of dehydroascorbate suggests high ascorbate content in *E*. *huxleyi* cells. It should be noted that a certain amount of threonate, which is known to be a major product of ascorbate degradation [25], was also detected. These suggest that ascorbate plays an important antioxidative role in *E*. *huxleyi*. Alpha-tocopherol, known as a type of vitamin E, was detected and might also have antioxidant function [26].

#### 2.2.3. Low Contents of Malate and Fumarate

Malate and fumarate are intermediates of tricarboxylic acid (TCA) cycle but also considered to have various other functions in plant cells. Malate is likely present in all cell types and can accumulate to levels as high as 350 mM [27]. It has been proposed to exhibit multiplicity of functions in many processes including C_4_ and CAM photosynthesis, carbon storage, pH regulation and nutrient uptake [28]. Fumarate appears to behave as both a temporary carbon sink for photosynthate similar to starch, and as a pH regulator in nitrate assimilation in green plants [29]. The amount of carbon accumulated in fumarate can be similar to that accumulated in starch under certain growth conditions [29,30]. It can accumulate to several milligrams per gram fresh weight of leaf tissue, which equates to concentrations up to 50 mM, depending on its location in the cell [30]. Whilst these metabolites represent most prominent peaks in metabolite profiling of most plant samples, neither malate nor fumarate was detected in *E*. *huxleyi* cells (Table 1). This suggests that the contents of these metabolites are quite low and the central carbon metabolism of this alga is quite distinct from that of green plants. Given that the production of these metabolites is closely related to tricarboxylic acid (TCA) cycle activity, *E*. *huxleyi* likely are far less reliant on the TCA cycle as has previously been documented for cyanobacteria and diatom [4,31,32,33]. The functions of malate and fumarate in land plants may partly be mediated by high accumulation of mannitol in *E*. *huxleyi*, which can also function in carbon storage and redox regulation [20,21].

### 2.3. _13_C-Label Accumulation Analysis Suggested Large Metabolic Flux into C_3_ Pathway and Mannitol Synthesis

**Figure 2:**
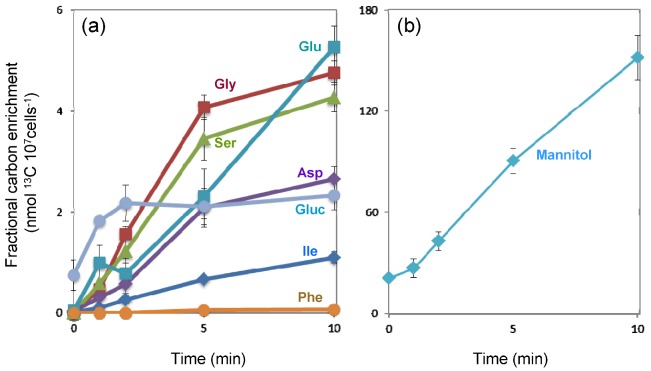
Time course of net ^13^C-isotope incorporation into the metabolites during 10 min of H^13^CO_3_^−^ feeding. (a) glutamate, blue green square; glycine, red square; serine, green triangle; aspartate, purple diamond; glucose, blue gray circle; isoleucine, blue diamond; phenylalanine, orange circle. Mannitol is plotted separately in (b) on a different scale due to much higher values than other metabolites. Values are means of nmol ^13^C-incorporation into mass fragments ± standard error of means from four biological replicates.

The metabolite profile of *E*. *huxleyi* suggested a distinctive central carbon metabolism with an essential role of mannitol. To further evaluate the function of mannitol and to gain an overview of metabolic flux from CO_2_ fixation, ^13^C label accumulation analysis was conducted using H^13^CO_3_^−^ as a substrate. The accumulation of ^13^C-labeled fragment in each metabolite was analyzed at 0, 1, 2, 5 and 10 min after the addition of NaH^13^CO_3_ into the culture. ^13^C incorporation was detected in eleven metabolites including amino acids and sugars (Figure 1). Absolute amounts of selected metabolites from each chemical class were quantified and the net ^13^C-incorporation into those metabolites was calculated (Figure 2). The results suggested flux through the C_3_ pathway of carbon assimilation and a large mannitol biosynthetic flux as discussed below.

#### 2.3.1. The C_3_ Cycle is Dominant for CO_2_ Fixation in *E. huxleyi*

**Figure 3:**
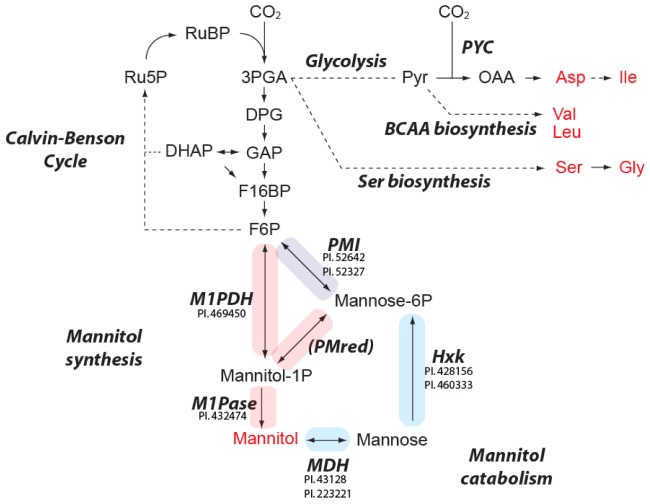
Overview of the CO_2_ fixation pathway and predicted enzymes for mannitol metabolism in *E*. *huxleyi*. Regular and bold italic indicate metabolites and enzymes or metabolic pathways, respectively. Metabolites in red were detected in the current study. Other metabolites except for pyruvate and mannose are hardly detected by GC-MS analysis. The proteins showing significant homology to known enzymes are listed close to the enzyme name. Protein IDs are taken from JGI *Emiliania huxleyi* CCMP1516 main genome assembly v1.0. Solid and dashed arrows show single and multiple reactions, respectively. The reactions colored by red, blue and purple are related to manntiol biosynthesis, catabolism and both pathways, respectively. Ru5P, riburose-5-phosphate; RuBP, riburose bisphosphate; 3PGA, 3-phosphoglycerate; DPG, 1,3-diphosphoglycerate; GAP, glyceraldehyd-3-phosphate; DHAP, dihydroxyacetone phosphate; F16BP, fructose-1,6-bisphosphate; F6P, fructose-6-phosphate; Pyr, pyruvate; OAA, oxaloacetate; BCAA, branched chain amino acid; PYC, pyruvate carboxylase; PMI, phosphomannose isomerase; PMred, phosphomannose reductase; M1PDH, mannitol-1-phosphate dehydrogenase; M1Pase, mannose-1-phosphatate; MDH, mannitol dehydrogenase; Hxk, hexokinase.

^13^C accumulated very rapidly in aspartate and serine within the first 5 min of incubation. Glycine also displayed a similar pattern of accumulation but it exhibited a lag period of 1 min before the label started to accumulate. After 5 min of feeding the label accumulation showed a saturating tendency in these amino acids (Figure 1). A very rapid label accumulation into aspartate has previously been reported and attributed to the contribution of β-carboxylating enzymes, namely pyruvate carboxylase (PYC), phospho*enol*pyruvate carboxylase (PEPC) and phospho*enol*pyruvate carboxykinase (PEPCK) [12]. However in the current study, the label accumulation patterns in aspartate and serine were quite similar (Figure 1). Since these metabolites share 3-phosphoglycerate (3PGA) as a common precursor, it is thus reasonable to assume that the majority of the fixed carbon came through the C_3_ cycle via 3PGA (Figure 3). This confirms the previous observations that the dominant initial photosynthates were phosphoesters in *E*. *huxleyi* [12]. It should be emphasized that there is no doubt regarding the involvement of β-carboxylation since there is genetic and enzymatic evidence supporting this [12,13], however, it appears to be quantitatively minor in comparison to the flux passing through the C_3_ cycle. Very recently the results of ^13^CO_2_-labeling analysis in *Arabidopsis thaliana* rosettes were published [34]. Aspartate and serine showed label accumulation kinetics distinct from *E*. *huxleyi*. The label accumulation saturated in serine whereas that in aspartate increased over 60 min following 2 min of lag period [34]. Such contrasts between these two organisms likely reflect the activities of the pathways downstream of 3PGA. High flux through the glycolytic pathway is apparent in *E*. *huxleyi*, since aspartate, which is synthesized from pyruvate, was quickly labeled. The label accumulation into serine in *E*. *huxleyi* showed a very short lag suggesting the contribution of the serine biosynthetic pathway from 3PGA involving just three enzyme reactions from CO_2_ fixation rather than that of the photorespiratory pathway. On the other hand, photorespiratory flux is likely to be responsible for serine biosynthesis in Arabidopsis since photorespiratory flux is quite high in C_3_ plants [35]. However, future experiments will be required to clarify this.

#### 2.3.2. Extremely High Flux into Mannitol

In contrast to glucose and *myo*inositol, in which label accumulation saturated within a few minutes, the label in mannitol linearly increased over time following a one-minute lag period (Figure 1). While the ratio of labeled mass fragment was only 0.2 to 2% due to a large pool of unlabeled mannitol, fractional carbon enrichment, which reflects the amount of labeled carbon accumulated in a metabolite, is much higher than that of other metabolites (Figure 2). A certain amount of carbon is likely to be fixed as methanol insoluble glucans and lipids that cannot be detected with our methods [12]. Nonetheless, our observation suggests that the large fraction of fixed carbon is accumulated as mannitol at least in this growth condition. Thus together with the high cellular concentration, mannitol most likely serve as storage compound as reported in certain algae and green plants [19,36,37]. In some unicellular algae including *E*. *huxleyi* and diatoms, lipids and glucans have been considered as main storage compounds [38,39]. ^14^C-labelling studies have revealed a carbon flux from phosphoesters to these compounds in *E*. *huxleyi* [12]. The current results suggest mannitol as additional carbon storage form in this alga. Several advantages have been implied for photosynthetic organisms that use mannitol as a photoassimilate. It has been hypothesized to allow a high NADP/NADPH turnover rate resulting in high photosynthetic activity since cytosolic mannitol can serve as an additional sink of reducing equivalent other than glucans [40]. Actually high photosynthetic rates, equivalent to those of many C_4_ plants, are observed in celery which has a typical C_3_ anatomy and uses mannitol as storage compound [40]. A second potential advantage of mannitol metabolism is the lower energy cost for remobilization than glucans and lipids. Additionally, NADH production in the first step of mannitol catabolism mediated by mannitol dehydrogenase (MDH, Figure 3) allows efficient release of reducing equivalents [41].

It is of particular interest whether mannitol is related to a storage glucan in *E*. *huxleyi*. *E*. *huxleyi* accumulates a β-polyglucan consisting of glucose polymers linked through β-1,3 and β-1,6 linkages [42]. The metabolism of β-polyglucan is poorly studied as opposed to that of α-polyglucans such as starch and glycogen. However laminarin, another storage β-polyglucan found in brown alga has been the subject of at least some studies. Radiolabeling experiments have demonstrated that laminarin and mannitol represent major parts of carbon storage and that they are interchangeable in brown algae, as are sucrose and starch in higher plants [43]*.* A genomic study of *Ectocarpus siliculosus* suggested a GT48 family β-1,3-glucan synthase as a candidate enzyme involved in addition of mannitol onto the end of polyglucan chain [36]. However the β-polyglucan in *E*. *huxleyi* consists of more than 99% glucose and only trace amount of mannitol residues in contrast to laminarin which terminates in 1-linked mannitol residues [42,44]. Further studies are, however, required to reveal the biosynthetic pathway of the storage glucan and interchangeability of mannitol with it.

### 2.4. Predicted Pathways for Mannitol Metabolism in *E. huxleyi*

Biosynthesis of mannitol is well studied in celery and brown algae, both of which synthesize mannitol as a major storage carbohydrate. Mannitol is produced in an amount approximately equivalent to sucrose via the intermediates mannose-6-phosphate (Mannose-6P) and mannitol-1-phosphate (M1P) in celery leaves [19]. Fructose-6-phosphate (F6P) produced by Calvin-Benson cycle is converted to Mannose-6P by phosphomannose isomerase (PMI) and then to M1P by phosphomannose reductase (PMred). M1P is then dephosphorylated by mannitol-1-phosphatase (M1Pase) to form mannitol [20]. In the sequenced brown alga *Ectocarpus siliculosus,* manitol is the most abundant solute and its level shows diurnal oscillation [45]. In this alga F6P is hydrated by mannitol-1-phosphate dehydrogenase (M1PDH) to produce M1P. To evaluate if these pathways are operative, the homologs of these enzymes were searched in JGI *Emiliania huxleyi* CCMP1516 main genome assembly v1.0 [17]. Interestingly, homologs of all proteins involved in both pathways with the exception of PMred were found (Figure 3). Whilst more than 50 proteins showed significant homology with known PMred proteins, it was difficult to identify PMred genes due to the high homology with other oxidoreductases. On the other hand no homologous proteins to the enzymes involved in starch and sucrose synthesis in Arabidopsis (starch synthases, sucrose synthases, sucrose phosphatases and sucrose phosphate synthases) were detected albeit the genomic sequencing of *E*. *huxleyi* is not yet completed. This is in accordance with our metabolite profiling results showing high accumulation of mannitol but relatively low levels of sucrose (Table 1). Accumulated mannitol could be re-mobilized by reduction to mannose by mannitol dehydrogenase (MDH) (PI.43128 and PI.223221) and phosphorylation to Mannose-6P by hexokinase (Hxk) (PI.428156 and PI.460333). PMI can also convert mannose-6P to F6P, which is involved in many metabolic pathways including glycolysis. It should be noted that PI.469450 showed similarity to both M1PDH and mannitol 2-dehydrogenase (M2DH). It is difficult to distinguish these enzymes by amino acid sequence since they are evolutionary related and thus highly homologous [46]. If the PI.469450 protein catalyzes the M2DH reaction, mannitol can also be dehydrated to fructose and then phosphorylated to F6P by Hxk. The genomic sequence of a brown alga *E*. *siliculosus* also contains no sucrose and starch synthetic enzymes but codes a set of enzymes for mannitol metabolism [36]. It also displays a similar metabolite profile to *E*. *huxleyi,* characterized by a very low amount of sucrose and accumulation of mannitol [45]. However the components of mannitol metabolism are slightly different from those of *E*. *huxleyi*. *E*. *siliculosus* possesses distinctive M1PDH found only in few organisms including brown alga and Apicomplexa. Apicomplexan M1Pase is only one M1Pase protein, which has been biochemically identified so far, but the *E*. *siliculosus* genome lacks the gene encoding a homolog of this protein. It also lacks a broad-specificity Hxk, which is typical of multicellular eukaryotes. This alga should thus use M2DH to produce fructose and fructose-specific kinase to remobilize mannitol [36]. In contrast, *E*. *huxleyi* has M1Pase and Hxk, which are highly homologous to Apicomplexa and plant enzymes, respectively. However it possesses no M1PDH similar to brown algae (Figure 3). These results thus indicate diverse evolutional histories of algal metabolisms even if these converge to yield ultimately similar metabolite profiles.

## 3. Experimental

### 3.1. Algal Strain and Culture Condition

*Emiliania huxleyi* clone CCMP1516 was grown in autoclaved synthetic ocean water medium (Aquil*, recipe according to the National Center for Marine Algae and Microbiota (NCMA) [47], with modified concentration of nitrate (0.2 mM) and phosphate (0.01 µM)) at 18 °C and constant light at 30 µmol m^−2^ s^−1^. The medium contained 2.38 mM of NaHCO_3_ and the pH of the media was 8.05. Bacteria-free pre-cultures of CCMP1516 were prepared by supplementing the medium with 100 µg mL^−1^ streptomycin and 100 µg mL^−1^ betabactyl and used to inoculate antibiotic-free cultures for metabolite analysis. Prior to inoculation pre-cultures were microscopically checked for absence of bacterial contaminations by DAPI (4',6-diamidino-2-phenylindole) staining. Algal cell density and cell volume were determined using a Beckman Coulter Z2 particle counter and size analyzer. OD_750_ was measured by a spectrophotometer (Lambda25, Perkin Elmer, Waltham, MA, USA). Chl*a* content in cellular extract was spectrophotometrically determined by multiplying the absorbance at 665 nm with specific absorbance of 13.9 [48].

### 3.2. Sampling and Extraction Procedure for Metabolite Profiling

The cells in late logarithmic growth phase were harvested on a Durapore-HV membrane filter disk with 2.5 cm diameter and 0.45 µm pores (Millipore, Billerica, MA) by vacuum filtration. The filter with the cells was then transferred into a 1.5 ml tube and frozen in liquid nitrogen. The time from sampling to freezing the sample was about 20 s. Frozen samples were stored at −80 °C till metabolite extraction. Metabolites were extracted immersing the filter in 1 ml of 90% (v/v) methanol containing 0.1 µg mL^−1^ U-^13^C-sorbitol as an internal standard. Following 10 s of vortex, the filter was removed and the remaining solution was centrifuged at 22,000 × *g* for 5 min at 4 °C. A 50 µL aliquot of the supernatant was used for Chl*a* determination and 900 µL was dried by a vacuum concentrator (SpeedVac concentrator, Thermo, Waltham, MA). Dried samples were stored at −80 °C after filling the tubes with argon gas.

### 3.3. GC-MS Based Metabolite Profiling

Derivatization of metabolites and GC-MS analysis were performed as described in [49] using half of the volume of the solutions for derivatization. Chromatograms and mass spectra were evaluated by Chroma TOF^®^ 4.2 (Leco, St Joseph, MI) and TagFinder 4.0 [50]. Analytes were manually identified using TagFinder by comparing to the reference library mass spectra and retention indices in the Golm Metabolome Database [51]. The amount of metabolites was analyzed as relative metabolite abundance calculated by normalization of signal intensity to that of ^13^C-sorbitol which was added as an internal standard. Metabolite profiling data are reported following recent recommendations (Supplementary Table 2) [52]. Recombination experiments were carried out by assessing the recovery of *E*. *huxleyi* metabolites of defined quantity in a mixed alga-Arabidopsis (*Arabidopsis thaliana* Col-0 leaves) extract containing defined quantities of Arabidopsis metabolites.

### 3.4. H^13^CO_3_^−^ Feeding and Label Accumulation Analysis

The cells in late logarithmic growth phase were fed with NaH^13^CO_3_ (Sigma-Aldrich, St. Louis, MO) to gain 1 mM of final concentration for 0, 1, 2, 5 and 10 min under the same growth condition as stated above. The metabolites were extracted and analyzed by GC-MS as described above. The pH of the culture solution was checked by pH test paper and no significant shift of pH by the addition of NaHCO_3_ was observed. The ^13^C enrichment in each mass fragment was calculated as described in [53]. Absolute amounts of metabolites were determined using a standard curve constructed from the signal intensity of reference compounds with known amount.

### 3.5. Identification of Enzymes Involved in Mannitol Biosynthesis and Catabolism

The enzymes catalyzing the reactions of mannitol biosynthesis and catabolic pathways encoded by the *E*. *huxleyi* genome were predicted by homology with biochemically characterized proteins selected in the UniProt database. The amino acid query sequences were submitted to BLASTp search against “Emiliania huxleyi v1 best proteins” database on the JGI Genome Portal webpage [17] with a E-value cut off of 1.0e^−5^. The protein was considered as a candidate when the corresponding expressed sequence tags (ESTs) were available underpinning their expression. For each identified *Emiliania* protein, evidence of conserved protein modules was queried using the blastp tool in the NCBI database [54] to evaluate the existence of functional domains.

## 4. Conclusions

Here we describe the establishment and optimization of a protocol for GC-MS based metabolite profiling for the coccolithophorid alga *Emiliania huxleyi*. Our experiments revealed that simple cold methanol extraction of vacuum filtrated 10^7^ cells is expedient to explore the central carbon metabolism in this alga. The here established protocol extents the tool kit for *Emiliania* by a valuable technique and helps to better understand processes such as adaptation to environmental changes [55], phase switching events and calcification [56] in *Emiliania*. By following our protocol one can relatively easily apply GC-MS analysis to other algal species. Sampling of a large culture volume in a short time by vacuum filtration makes the analysis of algae, which cannot be grown to high cellular density in a laboratory culture, possible. The sampling method is also crucial to investigate algal physiology in a highly diluted culture which is similar to the natural habitat given that microalgae live in much less density in the ocean than in a laboratory culture. The first application of this protocol demonstrated that the CO_2_ fixation pathway in *E*. *huxleyi* was characterized by a high metabolic flux from C_3_ cycle into mannitol suggesting its role in carbon storage. The additional energy storage as mannitol and efficient conservation of reducing equivalent by mannitol metabolism [40,41] might be the key factors responsible for the ecological success of *Emiliania* in current oceans. However, the metabolite profile should be repeatedly tested in various growth conditions to confirm the storage function of mannitol in a natural environment given the stress-related nature of mannitol and unnatural environment in the batch culture, which might cause accumulation of mannitol. The activities and properties of key enzymes in mannitol metabolism should also be tested in the future to provide proof of their functionality and to reveal the regulatory mechanism of these important pathways.

## Supplementary Files

Supplementary File 1 Supplementary Information (PDF, 21 KB)

Supplementary File 2 Supplemental Table (XLS, 46 KB)
