# Supplementary material for: Gas-Chromatography Mass-Spectrometry (GC-MS) Based Metabolite Profiling Reveals Mannitol as a Major Storage Carbohydrate in the Coccolithophorid Alga Emiliania huxleyi
Source: Metabolites. 2013 Mar 11;3(1):168–84. doi: 10.3390/metabo3010168 (PMC3901260; doi:10.3390/metabo3010168)
Supplement: Supplementary File 1 — Supplementary Information (PDF, 21 KB) [file metabolites-03-00168-s001.pdf]

Supplementary Information

Figure 1. Optimization of extraction procedure. (**a**) Effect of incubation time on the efficiency of metabolite extraction. Following the addition of 90% methanol and vortex, cells are incubated at 4°C for 30 min or 60 min with shake. The amounts of extracted chlorophyll *a* are determined as an indicator of metabolite extraction. (**b**) Effect of sonication. The samples are sonicated for 3 min after vortex and incubated for 60 min. In both experiments 5.8 x 10^6 cells are extracted with 2.0 ml of methanol solution. Values are means ± SEM from 4 technical replicates. Asterisk indicates the condition showed statistically significant difference from vortexed samples by *t*-test (p<0.05).

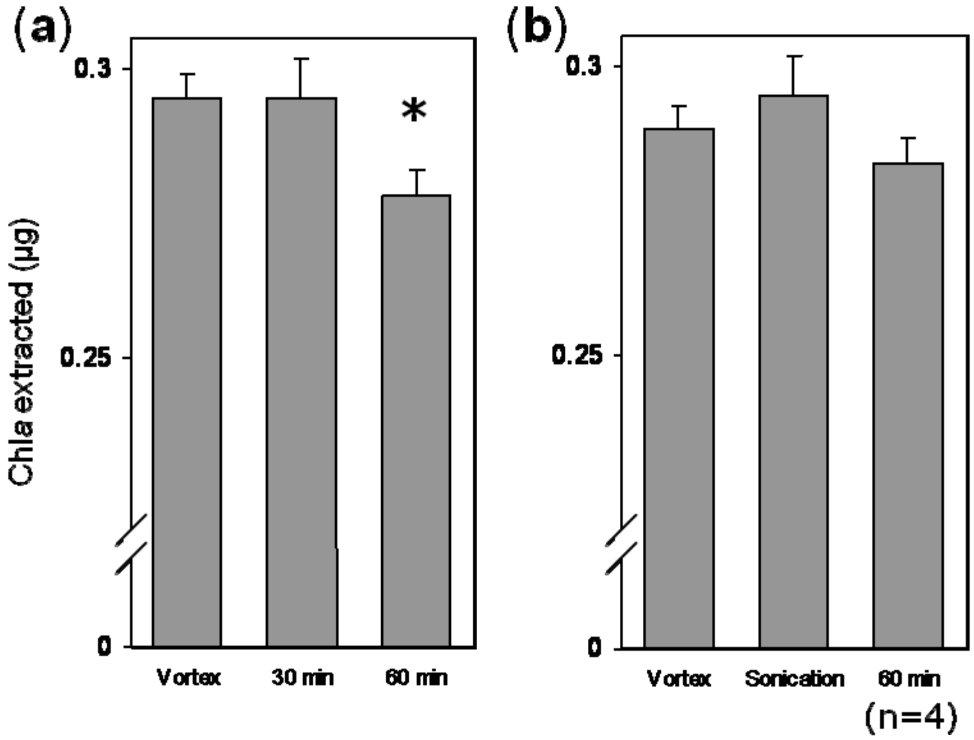

Table 1. Number of metabolite detected from different number of cells.

Cell number (x10^6 cells)	1.16	2.32	5.80	11.6	23.2
Number of detected metabolites	2	9	17	26	26
Number of overloaded metabolites	0	0	0	0	2
